# Does financial support for medical students from low income families make a difference? A qualitative evaluation

**DOI:** 10.1186/s12909-019-1573-3

**Published:** 2019-05-17

**Authors:** Hugh Claridge, Michael Ussher

**Affiliations:** 1 Population Health Research Institute, St George’s, University of London, Cranmer Terrace, London, SW17 0RE UK; 2 Institute for Social Marketing, University of Stirling, Stirling, FK8 1NZ UK

**Keywords:** Financial support, Bursary, Undergraduate medical student, University, Qualitative research

## Abstract

**Background:**

The 2015–2020 strategic plan from the Office for Fair Access calls on institutions to provide contemporary assessments of the impact of their financial support for disadvantaged students on retention, progression, success, wellbeing and participation, throughout the student lifecycle. In response to this call, this article describes the first evaluation the authors are aware of, of a financial support scheme for students from lower income backgrounds attending a medical school.

**Methods:**

A qualitative study of a bursary scheme for undergraduate medical students was undertaken at a university in London, England. One-to-one, audio-recorded interviews were conducted, transcribed and thematically analysed in order to ascertain eight recipients’ experiences of receiving the bursary and its influence on their financial situation, academic studies and quality of life.

**Results:**

The data were best explained by five main themes: impact of the bursary, communication, financial management, support preferences, and administration of the bursary.

**Conclusions:**

The participants, who were in receipt of various bursary amounts, generally regarded it as a good scheme with it providing a financial buffer and enabling them to focus on their studies and extracurricular activities rather than seek paid employment during term time.

**Electronic supplementary material:**

The online version of this article (10.1186/s12909-019-1573-3) contains supplementary material, which is available to authorized users.

## Background

The English National Strategy for Access and Student Success [1] emphasises the need to improve the participation, retention and progression rates of higher education (HE) students from the most disadvantaged groups. It also highlights the need for evidence that financial support given to such disadvantaged students by higher education institutions (HEIs) is having a measureable and meaningful impact.

The evidence from previous research is mixed, with some studies suggesting that financial support positively impacts HE students’ academic performance [2] [3], wellbeing and retention [4], as well as influencing their university choice [5]. Other research has reported that such financial support has no observable effect on student retention [6] or university choice [7]. International research has found improved low-income student access and degree completion using performance-linked financial support [8].Significantly increased completion rates among doctoral neuroscience students with full financial support have also been found [9]. However, the applicability of international research findings are limited in this context due to the substantial differences found in HE situations across the globe, including tuition fee amounts, living costs and levels of public funding [10].

The National Strategy [1] is applicable to all undergraduate students, including those studying medicine, but the authors could not identify any evaluations of financial support specifically relating to undergraduate medical students in England. Medical students are distinct from students on other first undergraduate degree courses due to a combination of factors, including increased course length (5 versus 3 years’ study); increased semester length; and increased frequency of term-time travel due to medical placements. Furthermore, the evidence cited above may be of limited value because the data were collected before 2012–13. In 2012–13, places at HEIs switched from being predominantly publicly-funded to being funded by students, with annual tuition approximately trebling for most students to £9000 [1]. The government introduced several measures to help lessen the negative impact of this fee rise on the most disadvantaged students, such as the National Scholarship Programme (NSP) [11]; however, this was only implemented for the first 3 years after the fee increase. Considering the potential impact of the fee rise, the 2015–2020 strategic plan from the Office for Fair Access (OFFA) [12] calls on institutions to provide contemporary assessments of the impact of their financial support for disadvantaged students on retention, progression, success, wellbeing and participation, throughout the student lifecycle.

In response to this call, this article describes the first evaluation the authors are aware of, of a financial support scheme for students from lower income backgrounds attending medical school.

## Methods

### Aim

The aim of the study was to gather qualitative data, via interviews and focus groups, in order to assess the views of students at one London, England medical school who were in receipt of a means-tested bursary scheme, on their experiences of receiving the bursary and its influence (if any) on their financial situation, academic studies and quality of life.

### Design

A qualitative descriptive methodology was chosen as it allowed an in-depth and non-hypothesis-driven approach to eliciting a rich description of experiences and events relating to individual students [13]. A combination of focus groups and one-to-one interviews was chosen to allow participants to choose whichever they preferred, due the potentially sensitive nature of discussing personal financial information.

### Participants

A convenience sample of students was recruited via an email to all first year students on the 5 year Bachelor of Medicine, Bachelor of Surgery (MBBS) course at a medical school in London (hereafter referred to as ‘the University’), who were in receipt of the institution’s ‘Opportunity Fund Grant’. The grant aims to assist first undergraduate degree students from lower income backgrounds by providing non-repayable financial support in addition to their student finance, and ranges in amount from £300 to £2000 (the latter either being all cash, or £1000 accommodation fee waiver and £1000 cash), with the payments made biannually (see Additional file 1: Appendix 1 for eligibility criteria). The invitation provided contextual information for why they were being contacted and informed the participants that the research was being conducted to ascertain their thoughts and experiences of the bursary scheme and that their participation was entirely voluntary.

### Interview topic guide and procedure

A topic guide for the focus groups and semi-structured interviews with a combination of open-ended and closed questions, whilst not pilot tested, was developed by the research team in consultation with the University’s Student Finance and Widening Participation teams. The topics included: impact of the bursary on their lives; knowledge of financial support packages; sources of income; living costs and situation; changes they’d like made to the bursary scheme; and awareness of financial advice (see Additional file 2: Appendix 2 for the full Interview Topic Guide).

One researcher conducted the interviews (HC, male, master’s degree, researcher in public health, with experience of interviewing), who was not known to the students prior to their interview invitations and had no vested interest in the research topic. HC introduced himself as a university researcher entirely independent of the Opportunity Fund Grant staff and the interviewees were provided with an Information and Consent form, informing them of the purpose of the research, their freedom to leave at any time and the confidential nature of the interview. The one-to-one interviews took place in a private room in the University, were audio-recorded and transcribed verbatim by HC and an external transcriber and subsequently anonymised. HC also made field notes during the interviews. Transcripts were not returned to participants for comment or correction. It was intended that interviews would be conducted until data saturation was reached, whereby no new issues emerged in two consecutive interviews. On completion of the interview, interviewees were given a £25 gift voucher of their choice. The research was classed as an audit of the funding scheme by the University Research Governance Team; therefore, ethics approval was not required. The Consolidated Criteria for Reporting Qualitative Research (COREQ) tool was used to ensure comprehensive reporting of the methods and findings [14] (see Additional file 3: Appendix 3).

### Analysis

Thematic analysis was used to summarize and analyse the data [15]. This enabled the researchers to gain insight into the views and experiences of each participant, while also identifying both similarities and differences between participants. Thematic analysis was ongoing during the study [15]. Initial coding was undertaken independently by two researchers (HC, MU), who read and familiarised themselves with the transcripts and assigned initial codes, similar codes were grouped and combined to create themes. Themes were reviewed, refined and labelled through discussions (HC, MU) to ensure that they accurately reflected the data. As such, a hybrid of both deductive and inductive approaches was used, as the topic guide used to steer the interviews was based on findings of previous research into bursary schemes, whilst the open-ended nature of the questions enabled the participants’ own experiences to stimulate further discussion not necessarily included in the topic guide. Software was not used to aid the analysis.

## Results

**Fig. 1:**
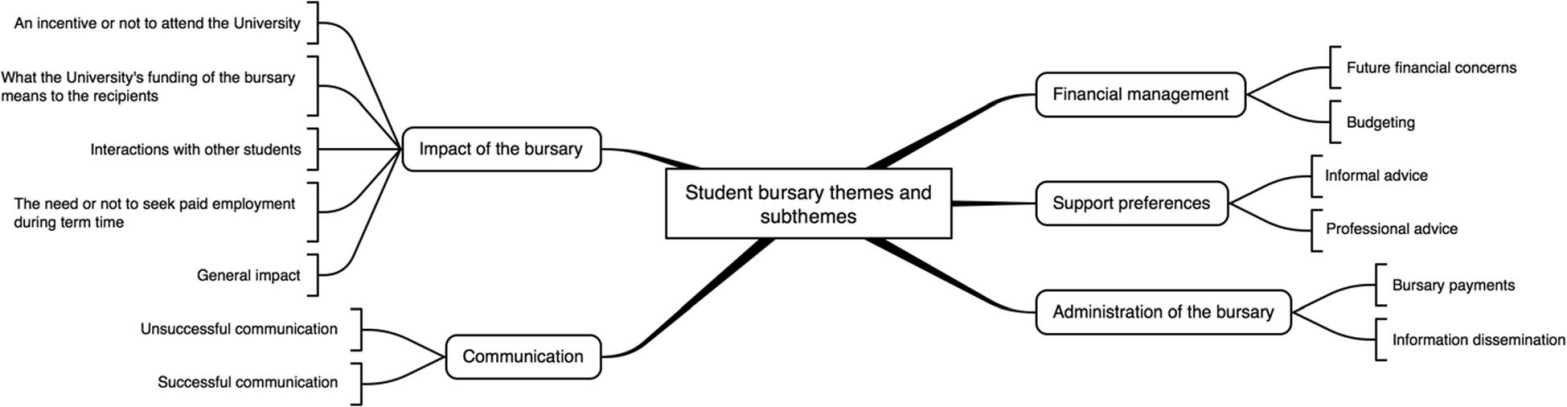
Spider diagram of themes and subthemes

Of 60 students invited to participate, 10 agreed to take part. Two of these were non-contactable and eight were interviewed in June 2016. It was not possible to arrange a focus group; therefore, interviews were used exclusively. There were four females and four males and interviews lasted for a mean (SD; range) of 33:06 min (19:46; 15:04 to 77:20). Five participants were in receipt of a £1000 accommodation fee waiver and £1000 cash, one received £2000 cash, one received £500 cash, and one received £400 cash. One was 19 years-of-age, five were 20, one was 21 and one was 22. To maintain their anonymity, due to the small sample size participant ethnicity cannot be provided. It was considered that data saturation had been achieved with eight interviews because with thematic analysis ongoing from the first interview onwards, we were able to note that no new issues arose in the final two interviews. After discussion among the research team, it was agreed that the data were best explained by five main themes, which are described below (see Fig. 1): (i) impact of the bursary, (ii) communication, (iii) financial management, (iv) support preferences, and (v) administration of the bursary.

### Theme 1: Impact of the bursary

#### Subtheme 1.1: An incentive or not to attend the University

No participant mentioned that the bursary had influenced their decision to enter higher education, and in several instances it could not have had any effect as some recipients were entirely unaware such a scheme existed before beginning their studies at the University. Therefore, opinions were mixed with regards to how the bursary influenced their choice of institution:

*Int3: Erm, no, I didn’t. No, that wasn’t a deciding factor.*

*Int8: They all had similar kind of things of varying amounts, but I wouldn’t say it was an incentive to go anywhere.*

One participant regarded the level of financial support available elsewhere as potentially influencing their choice of institution, but in reality it came down to which one they received an offer from:

*Int2: [The bursary played a role] I think when comparing [study institution] to [other institution], [other institution] gave a lot more support but then obviously it depended on which one I was given a place…*

#### Subtheme 1.2: What the University‘s funding of the bursary means to the recipients

Not all recipients of the bursary knew that it was the University itself that provided the funds, however when informed, almost all expressed feeling positive about the institution because of it:

*Int5: It is a nice feeling that the university saw and that they recognised that you needed some help…so it’s quite nice and reassuring that [the university] cares about their students and the welfare of them.*

*Int7: I guess it’s really good that they’re interested in making sure that more people can access higher education…*

For one recipient, the fact that all universities they had applied to offered bursary schemes, rather lessened the impact:

*Int8: I think it’s nice of them but I don’t know … when I was applying to other universities, pretty much all of them had it, so I thought it was just a standard thing that most universities have for low income background students.*

#### Subtheme 1.3: Interactions with other students

The general perception was that being the recipient of a bursary made no difference to their interactions with other students, in terms of stigma:

*Int4: For me I think it doesn’t affect my social life, so I don’t think people look at me in a different way because I’m getting a bursary or anything stupid like that.*

#### Subtheme 1.4: The need or not to seek paid employment during term time

However, it was clear that for some, the bursary money meant they did not have to work during term time which could potentially impact upon not only their social life and thus interactions with other students, but also upon their time for academic study:

*Int5: The third year when you’re paying for a house, that kind of makes you want to go out there and earn money … you don’t want to be the person who doesn’t have enough to give for that … but I guess with the bursary, you don’t have to.*

However, this was not the case for all recipients:

*Int4: I’m worried about…renting a house and putting down deposits and all of that stuff and paying admin fees. I don’t know how I’m going to do it without the job! [slight laugh] So I have to get a job that’s why I’m a bit stressed about getting a job, that’s the thing.*

*Int3: Without the bursary, I’d still not seek employment, just because my Mum wants me to concentrate on it, like I should concentrate on my studying.*

#### Subtheme 1.5: General impact

The bursary money also enabled a student to take part in sporting activities that they otherwise may not have been able to:

*Int7: So going back to the rowing thing … it is quite expensive. Subs for first term were £15 but then the second term were £50 and then they were £75, and then on top of that you’re paying for travel…so it all adds up and I think I would have found it a lot harder if I didn’t have a bursary.*

Receiving the bursary was noted as being somewhat of a stress-reliever for not only some students, but also their parents:

*Int1: But this term, now that I’m having to sort of put deposits down on houses, it’s been quite a big help and I would have been quite stressed if I didn’t have it. But up until now, it was just sitting there, I didn’t even need it.*

*Int7: ...without the bursary, I think it would have been definitely a greater worry for my Dad and stuff.*

### Theme 2: Communication

#### Subtheme 1.1: Unsuccessful communication

The ability to successfully communicate the information relating to the bursary, such as its existence, the eligibility criteria and the total money involved, is likely to affect the impact of the bursary. Several participants mentioned only finding out there was a bursary when they were told they would be receiving it:

*Int6: No, I had no idea, because when I got the letter about it, I had no idea about it and I still don’t know what it’s for, like why I’d get it.*

*Int8: I remember we didn’t hear from them for a while, we weren’t sure when we were going to get it.*

For those aware of the bursary prior to beginning their studies at the University, some were concerned that they had heard nothing about it since arriving, and it was affecting their ability to budget appropriately:

*Int7: I would have liked to have heard something when I started, to be honest, not six weeks’ in or whatever it was, because then you just know where you’re standing and you know how to budget and stuff.*

For this student and others, there was a feeling that the precise terms of the bursary were still not effectively communicated, as they only found out how much they were receiving and how the bursary was being applied to them when their rent was lower than expected:

*Int7: I remember paying for my accommodation and it was £1,000 lower and no-one had told me that, it had just come off. So I called them just to make sure that I was paying the correct amount and they were like, “Yeah, it was because of the bursary” But it would have been nice to have just … even if they’d just sent an email saying, “You’re only paying for this now”.*

Another student only realised the size and nature of their bursary during the research interview, and thought the money received was just their accommodation deposit being refunded early:

*Int1: My rent was noticeably cheaper … But I thought that was the university, so obviously you put down a deposit upon the accommodation, I thought that was the uni just taking that off.*

This poor communication regarding the terms of the bursary for this student had a direct impact on their accommodation for the next academic year and their need to find a job over the summer break, as they were unaware of how much money they were receiving:

*“Int1: It would have…influenced my plans as to sort of, which house I would have gone for [next] term, knowing that I would have had my £500 back, again, as it were, because I already thought I had it back … and that sort of takes the heat off me earning as much this summer…”*

#### Subtheme 1.2: Successful communication

Clearly, some communication regarding the scheme was successful as some students were aware of it prior to arriving:

*Int7: ...on the Open day, one of the things that they talked about was the bursary so I was like, “Oh, yeah, this is really good”.*

Some students acknowledged that not being aware of the bursary scheme’s details may have been down to them:

*Int4: I wouldn’t say lack [of communication from the university], I would say more on my part, I wasn’t really looking for that aspect, because I kind of knew that I’d have Student Finance and that was it. So any time it would have something that’s not concerning me, I wouldn’t read it. So I’m sure it came up multiple times “Student Bursary this, available for that”.*

### Theme 3: Financial management

#### Subtheme 3.1: Future financial concerns

One of the unique aspects of studying undergraduate medicine is the extensive length of the course at 5 years. This brings about specific financial considerations for undergraduate medical students, especially given the option of intercalating – where students take time away from their primary course and study for another academic degree. For some of the bursary recipients, the monetary implications of intercalating was already on their minds:

*Int2: Finances is making me iffy about it…*

*Int3: … if I want to intercalate at a London uni, then I’d have to pay for even more central London accommodation kind of thing.*

But for others, whilst the financial aspect was a consideration, it was not the primary deterrent:

*Int5: But more than just finance, the reason I will not go for intercalation, if I have the opportunity, would be more sort of “I want to stabilise and get a job” and stuff, more than “£9,000 extra is going to be too much”, kind of thing.*

Many of the students were not aware of what would happen to their bursary payments in their second year, meaning they were unable to plan ahead for the next year’s costs:

*Int5: I think I might get £1,000, something like that. I don’t know because they haven’t emailed me so I can’t really say.*

#### Subtheme 3.2: Budgeting

Other research has found that some students have turned to ‘payday loan’ companies and gambling in order to boost their finances whilst at university [16], however no interviewee mentioned these as having been a consideration at this stage in their studies:

*Int5: So, no, I’ve never felt like I needed to go into that kind of thing, or a loan or anything. Because I was trying to save myself from an overdraft; I would rather get an overdraft than a loan*.

Several students raised their lack of planning for the costs associated with renting private accommodation as being a significant concern for them as they were unaware of the need to have cash deposits:

*Int1: But, now deposits which are a lot larger than the hall’s deposits were, so I thought “Oh my god” like, you know “It’s monstrous”*

Some were having to consider turning to ‘the bank of mum and dad’ to cover the shortfall:

*Int4: Yeah, I didn’t foresee that [deposit] and that was a problem. I don’t know how I’m going to deal with that! [nervous laugh] I will probably just have to go to my parents, because I don’t have any other way of getting money.*

Indeed, one of the above students had not told their parents they were in receipt of the bursary, precisely so that they would still be able to ask for money:

*Int4: Yeah, I didn’t tell them about the bursary, because it came in and I was like “If I tell them about the bursary, then they’ll think that I’ve got a lot of money and then they won’t give me any money”.*

### Theme 4: Support preferences

#### Subtheme 4.1: Informal advice

None of the participants sought financial advice or other related support from the University, and the responses were mixed with regards to whom they would turn if they ever needed such advice, with the majority preferring to consult family and friends first:

*Int1: …I think first and foremost I wouldn’t go to the university, I’d go to like family or friends first … only if I was like really hard up, I was in a complete mess, would I probably turn to the university.*

After family and friends, the preference for the nature of the University’s support services was having a fellow student there to give advice:

*Int4: …maybe a fellow student, because you’re more able to relate with a fellow student than a member of staff. So you’re able to ask them what they went through and stuff like that…*

#### Subtheme 4.2: Professional advice

One student did prefer the idea of a professional adviser rather than experienced peer, but the lack of awareness of the current support provider was also noted:

*Int7: I’d rather have someone who’s appointed to do this kind of thing. I’m sure there probably is, but we don’t really know who it is, if you know what I mean. It’s all a bit faceless and via email.*

### Theme: Administration of the bursary

#### Subtheme 5.1: Bursary payments

Aside from the communication issues raised above, most were satisfied with the general administration of the bursary, such as how the bursary payments were split over the year and how the accommodation fee waiver was applied:

*Int4: …two [payments] is better, because I came in and most of my grant was given to me at the start, and then after the grant was gone, then the bursary came in and that was good. If it was spread out even more then it would kind of like vanish, it’d disappear and it wouldn’t be that significant, if you know what I mean.*

Some expressed a preference for having the first bursary payment come earlier in the year, as they felt they needed a bit more of a financial buffer at that point, having only just started understanding budgeting and had spent an unusual amount due to it being the first term:

*Int8: I think it would have been slightly better if we could have got it earlier on in the year … I feel most students, including myself, are finding their feet with their own budgeting at the very beginning. So say you slightly overspend, you have that little bit of a safety net at the very beginning.*

#### Subtheme 5.2: Information dissemination

Some general recommendations were also made by the students, and these mainly related to how to improve the dissemination of information about the bursary and other financial support available:

*Int3: Maybe like a leaflet or in the SU if it has like financial help and stuff like that, something about budgeting and stuff for students and that.*

*Int4: A better way of contacting students I think would be to go through their Student Reps, those guys, because students actually listen to them and what they have to say on Facebook … it would reach the people that would need it and they’d see it…’*

## Discussion

The eight participants, who were in receipt of various bursary amounts, generally regarded it as a good scheme with it providing a financial buffer and enabling them to focus on their studies and extracurricular activities rather than seek paid employment during term time. As found by research into the NSP both before the 2012 fee rise [17] and after [18], the bursary did not act as an incentive to attend the University; however, this was not always due to the bursary itself but sometimes because their attendance depended on which university accepted their application or because they were not aware the bursary existed. Indeed, previous research into the NSP also found that the majority of potential scholarship holders only find out that they will receive an award and how much it includes after they have enrolled at their chosen university, meaning it cannot impact university choice [19]. Recipients of this University’s bursary only find out they qualify when Student Finance England has finished identifying eligible students, and this is completed weeks after arrival. This delay in finding out whether they qualify, combined with the communication issues raised by many participants, affected some participants’ ability to budget effectively. When informed that the University funded the bursary, most participants expressed gratitude and felt positively towards the University, echoing pre-fee rise findings [20]. The fact that a student did not wish their parents to know about the bursary scheme suggests that the University’s term-time communication regarding the bursary might be best only sent to their term-time address. The wellbeing of some recipients was reported to be positively impacted through reduced financial stress, as found by previous research [19]. However, again echoing past findings [18], reducing bursary amounts in future years and the need to pay significant cash deposits for accommodation were mentioned as stressors. The potential financial strain of an additional year’s fees brought about by intercalating was a concern for some, however it was clear that it was not of immediate consideration for most due to only being their first year studying. For similar reasons, the financial implications of travelling to medical placements in later years was already a concern for some, but not yet a consideration for others. Financial advice appeared to be preferred when given by friends and family rather than professionals employed by the University, although no participant had yet experienced such professional help.

As far as the authors are aware, this is the first example in the literature of qualitative research involving a bursary scheme for undergraduate medical students in England. It also responds to OFFA’s call for institutions to assess their financial support packages’ impact on recipients [12], whilst adding to the literature relating to qualitative evaluations of bursary schemes in the post-2012 system of student fees. Further strengths of this study include the use of semi-structured, one-to-one interviews led by a single interviewer, who was independent of both teaching and bursary processes, with recipients of a variety of bursary amounts and from a range of ethnicities. This enabled in-depth exploration of the subjects covered by the topic guide which was composed of both open-ended and closed questions, having been developed by both the research team and the University’s Student Finance and Widening Participation teams.

There were also limitations. The participants were from just one medical school in England, and the small number of first year students of medicine in receipt of the bursary meant it was not possible to perform meaningful statistical analysis on retention rates and attainment. It also limits the reach of the discussion in terms of the actual financial impact that intercalation and travelling to medical placements have on bursary recipients due to only first year students being involved. Furthermore, participant demographic information could not be provided with their quotes as this would jeopardise their anonymity due to the small number of bursary recipients. However, this group was chosen because we sought to determine the views of students who had only recently begun receiving the bursary, thereby reducing the potential for post-hoc rationalisation by those who had been in receipt of the bursary for longer [21]. We were also unable to recruit recipients of the £300 and £600 bursaries, nor could a focus group be arranged for willing participants. However, recipients of all other bursary amounts were represented and the one-to-one interviews likely allowed deeper exploration of individuals’ experiences than would have been possible in a group setting.

## Conclusions

This article documents the first qualitative study into a bursary scheme for medical students in England. The findings are broadly similar to those of previous research conducted among pre- and post-2012 fee rise students, in terms of the generally positive impact on the recipients’ wellbeing and ability to study rather than seek term-time employment, although the specific issues relating to medical students such as intercalation and medical placements, need to be explored further. However, this means that the findings presented here are likely to be applicable to bursary recipients studying almost any other full time undergraduate course in England as the medical students involved in this study have yet to experience the aforementioned unique aspects of their chosen course of study. Several participants planned to undertake paid work over the summer break and as this is unlikely to be possible in later years due to the shortened holiday periods for medical students, this highlights the potential for older years to be under increased financial strain and thus is worthy of research. Therefore, future research should involve bursary recipients in later years to evaluate the impact of not only the reducing financial support available compared with their first year of study and their reduced ability to work over the holidays, but also the additional costs of intercalation and travelling to medical placements. To limit the potential for post-hoc rationalisation, newer recipients of the bursary, but who are in later years of study, should be targeted. Comparing the impact the scheme has on those who live at home with family as opposed to living in student accommodation would also be of interest given these situations’ differing financial burdens.

## Additional files

Additional file 1: **Appendix 1.** Eligibility criteria for bursary from the University’s OFFA Access Agreement 2015/16. (DOCX 14 kb)

Additional file 2: **Appendix 2.** Interview Topic Guide. (DOCX 20 kb)

Additional file 3: **Appendix 3.** COREQ Checklist. (DOCX 19 kb)

## Abbreviations

COREQ: Consolidated Criteria for Reporting Qualitative Research
HC: Hugh Claridge
HE: Higher education
HEI: Higher education institution
MBBS: Bachelor of Medicine, Bachelor of Surgery
MU: Michael Ussher
NSP: National Scholarship Programme
OFFA: Office For Fair Access

## References

[1] Department for Business, Innovation and Skills (2014). National strategy for access and student success in higher education.

[2] Hatt S, Hannan A, Baxter A (2005). Bursaries and student success: a study of students from low-income groups at two institutions in the south west. High Educ Q.

[3] Murphy R, Wyness G (2016). Testing means-tested aid. Discussion paper.

[4] West A, Emmerson C, Frayne C, Hind A (2009). Examining the impact of opportunity bursaries on the financial circumstances and attitudes of undergraduate students in England. High Educ Q.

[5] Office for Fair Access (2009). Awareness, take-up and impact of institutional bursaries and scholarships in England: summary and recommendations.

[6] Office for Fair Access (2014). An interim report: do bursaries have an effect on retention rates?.

[7] Office for Fair Access (2010). Have bursaries influenced choices between universities?.

[8] Indiana Commission for Higher Education (2015). Reforming student financial aid to increase college completion: early Progress resulting from Indiana house enrolled act 1348.

[9] Miller A (2013). Timely doctoral completion rates in five fields: a two-part study. Graduate theses and dissertations.

[10] Organisation for Economic Co-operation and Development (2012). How are countries around the world supporting students in higher education? Education indicators in focus.

[11] Department for Business, Innovation and Skills (2011). The National Scholarship Programme: year one.

[12] Office for Fair Access (2015). Strategic plan 2015–2020. Plan.

[13] Neergaard MA, Olesen F, Andersen RS, Sondergaard J (2009). Qualitative description - the poor cousin of health research?. BMC Med Res Methodol.

[14] Tong A, Sainsbury P, Craig J (2007). Consolidated criteria for reporting qualitative research (COREQ): a 32-item checklist for interviews and focus groups. Int J Qual Health Care.

[15] Braun V, Clarke V (2006). Using thematic analysis in psychology. Qual Res Psychol.

[16] National Union of students (2012). The pound in your pocket: summary report.

[17] Callender C, Wilkinson D (2013). Student perceptions of the impact of bursaries and institutional aid on their higher education choices and the implications for the National Scholarship Programme in England. J Soc Policy.

[18] Bowes L, Moreton R, Thomas L, Sheen J, Birkin G, Richards S (2016). Evaluation of the National Scholarship Programme: year 4 report to HEFCE by CFE research and Edge Hill University.

[19] Bowes L, Moreton R, Thomas L, Porter A, Sheen J, Birkin G (2014). Evaluation of the National Scholarship Programme - year 3: report to HEFCE by CFE research and Edge Hill University.

[20] Harrison N, Baxter A, Hatt S (2007). From opportunity to OFFA: discretionary bursaries and their impact. J Access Policy Pract.

[21] Associates N (2015). What do we know about the evaluation of the impact of institutional financial support on access and success?.

