# Supplementary material for: Does financial support for medical students from low income families make a difference? A qualitative evaluation
Source: BMC Med Educ. 2019 May 17;19:153. doi: 10.1186/s12909-019-1573-3 (PMC6525429; doi:10.1186/s12909-019-1573-3)
Supplement: Supplementary file 1 — Appendix 1. Eligibility criteria for bursary from the University’s OFFA Access Agreement 2015/16. (DOCX 14 kb) [file 12909_2019_1573_MOESM1_ESM.docx]

Appendix 1 – Eligibility criteria for bursary from the University’s OFFA Access Agreement 2015/16

| Award offered | Priority Groups | Ineligible groups |
| --- | --- | --- |
| Total of £2000 for Year 1   - £1000 accommodation fee waiver (for university halls or additional £1000 cash if not in university halls) - £1000 cash award (2 x £500 over year)   Subsequent (non NHS  supported) years 2016/17  onwards cash awards between  £300-£1000 as in the bands  below | - Household income of £25,000 or less. - In receipt of maximum SFE maintenance grant. - Care leavers - Has attended institution’s WP Summer School and accepted institution as 1^st^ choice - UK English domiciled | - NHS funded students - Any other students whose fees are paid or part paid through a sponsorship arrangement - Students transferring into the institution’s undergraduate degree programme after completion of another programme - Students undertaking a post graduate qualification - 1st year graduate entry medical programme students (MBBS4) students |
| Band 5   - £1000 | - Household income of up to £25,000 |  |
| Band 4   - £600 | - Household income of £25,001 - £30,000 |  |
| Band 3   - £500 | - Household income of £30,001 - £35,000 |  |
| Band 2   - £400 | - Household income of £35,001 - £40,000 |  |
| Band 1   - £300 | - Household income of £42,001 - £42,600 |  |
