# Supplementary material for: Does financial support for medical students from low income families make a difference? A qualitative evaluation
Source: BMC Med Educ. 2019 May 17;19:153. doi: 10.1186/s12909-019-1573-3 (PMC6525429; doi:10.1186/s12909-019-1573-3)
Supplement: Supplementary file 2 — Appendix 2. Interview Topic Guide. (DOCX 20 kb) [file 12909_2019_1573_MOESM2_ESM.docx]

**Prior to coming to the University**

Did financial considerations play a role in your decision to go on to:

Higher education (HE)?

This University?

Study medicine?

Were you aware of students being able to obtain financial support from HE institutions prior to deciding to go on to HE?

If you weren’t aware, did you find out after deciding to go on to HE but before applying to universities?

If no again, did you only find out about financial support once you’d started applying?

Did the knowledge that you might be able to obtain a bursary influence:

Your decision to go on to higher education (HE)?

Your decision to study MBBS5?

Which HE institutions you applied to?

Your decision to study at this University?

How did you find out about this University’s bursaries?

Do you have any thoughts or suggestions about ways in which students should be informed about the University bursary?

**Current bursary situation**

Are you aware of where the bursary money comes from? [If yes, they need to say where to prevent them from pretending to know the answer]

If yes (being the University) do you feel your relationship with the University is any different to others who are not receiving a bursary? [This relies on them knowing the financial status of other students]

If no, now that you know it is the University, do you feel the same or differently towards it?

When would you like to know about whether you will receive the bursary and how much you will receive?

How would you prefer the bursary be supplied to you – fee waivers or cash?

How do you feel about the way the payments are spread out – are they fine as they are, or if not, when would you like them to be made and at what intervals?

Were you involved in the submission of your parents’ financial information to Student Finance England (SFE)?

How comfortable do you feel your parents were about sharing financial information with SFE?

Have you discussed your bursary with your parents, such as how much it is for and what it enables you to do that you wouldn’t be able to otherwise (if anything)?

How do you feel generally about the financial support package available to you from the University?

**Whilst at the University**

Do you feel that being a recipient of a bursary has influenced:

Your mentality towards your studies?

Your thoughts on whether to intercalate or not? (Are students aware that their SFE funding for the intercalated year is reduced due to the shorter academic terms of intercalated courses vs medicine?)

**Sources of income**

Are you aware of the University work opportunities available to you, such as working as a Student Ambassador, in the bar or school shop?

Do you earn money during term time?

Do you feel that being a recipient of a bursary has influenced whether you earn money, and if you do work, has it influenced the nature or amount of the work you do?

What sort of work do you do?

Why do you work during term time: money, experience, other?

Did you earn money by working prior to coming to the University?

Do you use any other means to ‘boost’ your finances, such as using credit cards, pay day loans, gambling etc.? If so, have you sought any financial advice prior to or whilst doing so?

**Living**

What are your living arrangements:

Whilst studying at the University?

During the University holidays?

Do you have any dependents (e.g. children, relatives or friends you provide care for)?

Do you feel you keep track of how much you spend each month on food, accommodation, educational items (text books, equipment etc.), gym, general ‘recreational’ costs/disposable income?

What do you feel are your ‘necessities’ as a student – food, accommodation, gym, going out etc.?

Have you encountered any unexpected costs in your time as a student thus far?

Do you feel that being a recipient of a bursary has influenced:

Your social life?

Your ability to partake in extra-curricular student activities?

Do you treat your bursary money differently to other income?

The financial circumstances of those studying at the University are varied – do you feel people’s financial backgrounds here have ever influenced your behaviour or the behaviour of others when studying/socialising/earning money etc.?

Do you have any persistent worries whilst studying at the University?

**Help**

A budgeting/finances presentation is given on the Taster Days, but would you also like a budgeting/financial presentation given at Induction Day?

Would you like a budgeting day that’s mandatory for all students, with associated workshops and talks? If yes, when and how often would you like them to be held?

How do you feel about the current type of professional budgeting advice given – do you like this method, or would you prefer for it to be a fellow student giving the advice?

A finance presentation is given at each Taster Day for offer holders. Do you think that students would benefit from additional timetabled finance advice sessions once they are enrolled on a course?

If yes, how would you like these sessions to be delivered (e.g. presentation, discussion group, workshops, etc.) and when do you think these should be held? Do you prefer advice sessions delivered by University staff/the Students’ Union/both? Are there any areas of financial concern that you think should be a focus?

If no, do you have any ideas about other format(s) of advice that might benefit students (we already offer one-to-one appointments, drop-in sessions, info sheets by email and in hard copy, and info online on the University Portal).

Do you feel you have any money troubles?
